# Supplementary material for: Higher Mutation Burden in High Proliferation Compartments of Heterogeneous Melanoma Tumors
Source: Int J Mol Sci. 2021 Apr 9;22(8):3886. doi: 10.3390/ijms22083886 (PMC8069012; doi:10.3390/ijms22083886)
Supplement: Supplementary file 1 [file ijms-22-03886-s001.pdf]

International Journal of Molecular Sciences

Supplementary Materials

Table S1. Main clinical and histopathological data of the eight patients excluded from the study.

Patient	Mel003	Mel004	Mel005a	Mel005b	Mel006	Mel007	Mel008	Mel009	Mel012
Sex	F	M	F	F	F	F	M	F	M
Age	59	66	85	85	88	65	76	84	88
Anatomical location	n.d.	Back	Left elbow	Left arm	Foot	Abdomen	Left temporoparieta l region	Cheek	Chest
Histological subtype	Epithelioid-fusocellular NM	Fusocellular NM	NM	LMM	Fusocellular NM	Amelanotic NM	NM	Fusocellular NM	NM
TNM	pT3b	pT4bN0	pT4bN0	pT1aN0	pT4bN2b	pT3b	pT3b	pT3a	pT4b
Clark	III	IV	n.d.		IV	IV	IV	IV	IV
Breslow	3.5 mm	4.5 mm	18 mm	<1 mm	8 mm	3 mm	22 mm	2.5 mm	9 mm
Ulceration	Yes	Yes	Yes	No	Yes	Yes	Yes	No	Yes
Mitotic index	5/mm^2	7/mm^2	2/mm^2	n.d.	13/mm^2	4/mm^2	1/mm^2	6/mm^2	25/mm^2
Lymphoid infiltration	Yes	Yes	Yes	n.d.	Yes	n.d.	No	Non-brisk	n.d.
Satellite tumors	No	No	No	n.d.	No	No	No	No	No
Lymph nodes	n.d.	Clear	Clear	n.d.	Metastases in 2/9	n.d.	n.d.	n.d.	n.d.
Ki-67	Homogeneous	Homogeneous	Homogeneous	Homogeneous	Homogeneous	Homogeneous	Homogeneous	Homogeneous	Homogeneous

HPF—high power field, LMM—Lentigo Maligna Melanoma, MF—mitotic figures, n.d.—no data, NM—nodular melanoma, SSM—superficial spreading melanoma.
